# Análise de Custo-Efetividade da Angiotomografia Coronária como Exame Preferencial na Investigação de Dor Torácica Estável na Saúde Suplementar no Brasil

**DOI:** 10.36660/abc.20250204

**Published:** 2026-01-09

**Authors:** Afonso Shiozaki, Jorge Torreão, Isabela Bispo Santos da Silva Costa, Anuncia Bouzas Suarez, Marcelo Tozatti da Silva, Thiago Godoy de Oliveira, Luana Emanuelly Sinhori Lopes, Marcelo Eidi Nita, Henrique Trad, Carlos E. Rochitte

**Affiliations:** 1 ND Núcleo Diagnóstico Maringá PR Brasil ND Núcleo Diagnóstico, Maringá, PR – Brasil; 2 Santa Casa de Misericórdia da Bahia Salvador BA Brasil Santa Casa de Misericórdia da Bahia, Salvador, BA – Brasil; 3 Instituto do Câncer do Estado de São Paulo São Paulo SP Brasil Instituto do Câncer do Estado de São Paulo, São Paulo, SP – Brasil; 4 Fundação Getúlio Vargas Rio de Janeiro RJ Brasil Fundação Getúlio Vargas,Rio de Janeiro, RJ – Brasil; 5 Universidade de Mogi das Cruzes Mogi das Cruzes SP Brasil Universidade de Mogi das Cruzes, Mogi das Cruzes, SP – Brasil; 6 Universidade Federal do ABC Santo André SP Brasil Universidade Federal do ABC, Santo André, SP – Brasil; 7 Universidade Estadual do Oeste do Paraná Cascavel PR Brasil Universidade Estadual do Oeste do Paraná, Cascavel, PR – Brasil; 8 Universidade de São Paulo São Paulo SP Brasil Universidade de São Paulo, São Paulo, SP – Brasil; 9 Lotus Radiologia Ribeirão Preto SP Brasil Lotus Radiologia, Ribeirão Preto, SP – Brasil; 10 Instituto do Coração do Hospital das Clínicas Faculdade de Medicina Universidade de São Paulo São Paulo SP Brasil Instituto do Coração do Hospital das Clínicas da Faculdade de Medicina da Universidade de São Paulo, São Paulo, SP – Brasil

**Keywords:** Tomografia Computadorizada por Raios X, Doença da Artéria Coronariana, Análise de Custo-Efetividade, Infarto do Miocárdio

## Abstract

**Fundamento:**

A doença cardiovascular é a principal causa de mortalidade mundial. Estratégias que priorizem o diagnóstico precoce podem reduzir a incidência de complicações e custo relacionados.

**Objetivo:**

Avaliar a custo-efetividade da angiotomografia coronariana (AngioTC) como estratégia de investigação inicial de dor torácica estável em pacientes com probabilidade pré-teste intermediária de doença arterial coronariana (DAC) estável, em comparação com a angiografia coronariana invasiva (ACI).

**Métodos:**

Foi realizada uma análise de custo-efetividade comparando AngioTC e ACI considerando dados da saúde suplementar brasileira. O modelo considerou os custos diretos dos exames diagnósticos, insumos, custo de internação por infarto e custo de revascularização miocárdica nas 5 regiões do Brasil. Foi realizada uma análise de impacto orçamentário referente à incorporação gradual da AngioTC ao longo de 5 anos, utilizando como população elegível um universo de 100.000 vidas.

**Resultados:**

A análise de custo-efetividade com AngioTC comparada à ACI, estimada para uma população de 100.000 vidas, mostrou uma economia de R$ 1.021,00 por vida ou de R$ 102.069.703,00 ao final do quinto ano. Quando considerado o valor médio da AngioTC por região, observamos, na população de 100.000, por vida e em 5 anos, respectivamente, uma economia média de: R$ 1.226,00 e R$ 122.577.793,00 na Região Norte; R$ 1.460,00 e R$ 145.988.367,00 na Região Nordeste; R$ 1.625,00 e R$ 162.502.626,00 na Região Centro-Oeste; R$ 1.313,00 e R$ 131.270.230,00 na Região Sudeste; e R$ 1.043,00 e R$ 104.268.937,00 na Região Sul.

**Conclusão:**

A AngioTC como estratégia inicial na investigação da dor torácica estável é custo-efetiva quando comparada à ACI e está associada à redução de custos significativos na saúde suplementar brasileira.

## Graphical abstract

**Figura Central:**
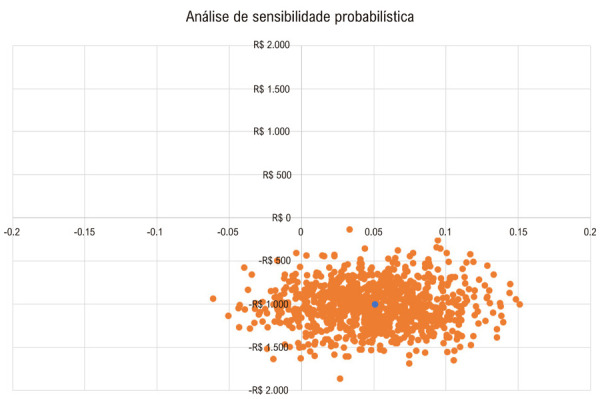
Análise de Custo-Efetividade da Angiotomografia Coronária como Exame Preferencial na Investigação de Dor Torácica Estável na Saúde Suplementar no Brasil

Análise de sensibilidade probabilística de custo-efetividade no cenário nacional. Nota-se que a grande maioria dos pontos (91,2%) caem sobre o quadrante inferior direito, que indica dominância em relação ao comparador. Apenas 8,8% dos pontos caem no quadrante inferior esquerdo de custo efetividade. Os pontos laranjas representam as 1.000 iterações probabilísticas feitas, já o ponto azul representa o ponto determinístico encontrado.

## Introdução

A doença cardiovascular é a principal causa de morte no mundo, maior do que todas as causas de câncer somadas, matando 3 vezes mais mulheres do que o câncer de mama e 7 vezes mais do que câncer de cólon.^1-3^ No Brasil, há mais de 350.000 mortes por ano, mais de 1.100 mortes por dia, cerca de 46 mortes por hora, 1 morte a cada 90 segundos.^4^ Diante dessa elevada carga de mortalidade, fornecer o diagnóstico precoce da doença arterial coronariana (DAC) torna-se essencial para redução de complicações relacionadas à ocorrência de eventos cardiovasculares maiores.

A DAC representa um impacto significativo tanto no sistema público de saúde quanto na economia nacional. Estima-se que, no Brasil, as doenças cardiovasculares gerem custos diretos e indiretos que ultrapassam R$ 50 bilhões por ano.^5^ Os gastos abrangem desde consultas, exames diagnósticos e terapias farmacológicas até procedimentos de alta complexidade, como angioplastia e cirurgia de revascularização miocárdica, que vem aumentado nos últimos anos no Brasil.^3^ Além disso, os custos indiretos relacionados à perda de produtividade, aposentadoria precoce e internações recorrentes acentuam ainda mais o ônus financeiro. Dessa forma, investir em estratégias diagnósticas que possam fornecer o diagnóstico precoce e preciso da DAC tem o potencial não apenas de melhorar o prognóstico clínico dos pacientes, mas também de mitigar possíveis custos a longo prazo, ao possibilitar intervenções oportunas que previnam desfechos adversos e hospitalizações evitáveis.

A estratégia diagnóstica da DAC é por vezes complexa, sendo discutida em várias situações clínicas de acordo com presença de sintomas e fatores de risco cardiovascular. A angiografia coronária invasiva (ACI) é o método padrão-ouro no diagnóstico anatômico da DAC.^6^ Entretanto, trata-se de um método invasivo, não isento de complicações relacionadas.^7^ A angiotomografia coronariana (AngioTC) é o único teste não invasivo capaz de visualizar a placa aterosclerótica coronariana, em diversas fases evolutivas, incluindo as não obstrutivas, permitindo a detecção e o tratamento mais precoce da DAC, reduzindo o número absoluto de infartos.^8,9^ Após a série de resultados do Ischemia Trial Group*,* parece consolidado o conceito de que a detecção e caracterização da carga aterosclerótica tem valor prognóstico superior à detecção de isquemia miocárdica.^10,11^ Diretrizes brasileiras atuais recomendam este exame como estratégia diagnóstica inicial em paciente com dor torácica, sem anatomia conhecida.^12^ Mas não há dados brasileiros que mostrem a custo-efetividade desta abordagem na saúde suplementar.

Diante disso, este estudo conduziu uma análise de custo-efetividade comparando as estratégias disponíveis para diagnóstico anatômico da DAC (AngioTC versus ACI) como procedimento inicial para avaliação de pacientes com angina estável no sistema de saúde suplementar. O objetivo foi estimar o potencial de redução de custos e o impacto clínico da adoção sistemática da AngioTC, com modelagem específica para diferentes regiões geográficas do Brasil, a fim de refletir variações nos custos e na infraestrutura disponível.

## Métodos

Foi realizada uma avaliação de custo-efetividade e de impacto orçamentário que levou em consideração a AngioTC como estratégia inicial na investigação da DAC em comparação à ACI. A escolha da ACI como comparador baseou-se no fato de que esta representa o método diagnóstico padrão-ouro na estratificação da DAC, sendo amplamente disponível no cenário nacional da saúde suplementar. Essa comparação se justifica pelo fato de a AngioTC ser o único método não invasivo disponível para o diagnóstico anatômico da DAC e com alta acurácia diagnóstica.^13^

Com a finalidade de aumentar a transparência do estudo proposto, os principais aspectos das análises foram sumarizados conforme o checklist Consolidated Health Economic Evaluation Reporting Standards (CHEERS), sob um horizonte de 5 anos, como recomendado pelo Ministério da Saúde.^14^

A análise focou na intervenção da AngioTC como diagnóstico inicial de pacientes com suspeita de DAC estável em comparação com a ACI. A população avaliada na modelagem corresponde a uma demanda simulada de uma operadora de saúde de médio porte, estimada em uma coorte anual de 100.000 beneficiários. Adicionalmente, foi aplicada à projeção populacional a taxa de crescimento demográfico anual definida pelo Instituto Brasileiro de Geografia e Estatística (IBGE).

A avaliação econômica foi conduzida com base em valores praticados no sistema de saúde suplementar brasileiro, utilizando como referência a Classificação Brasileira Hierarquizada de Procedimentos Médicos (CBHPM), elaborada pela Associação Médica Brasileira (AMB) em conjunto com as sociedades de especialidades médicas, com apoio técnico da Fundação Instituto de Pesquisas Econômicas (FIPE). De modo complementar, utilizou-se a lista oficial de preços de medicamentos publicada pela Câmara de Regulação do Mercado de Medicamentos (CMED), órgão interministerial composto por representantes dos Ministérios da Saúde, da Economia, da Justiça e da Casa Civil, sendo a Agência Nacional de Vigilância Sanitária (Anvisa) responsável por sua Secretaria-Executiva.^15,16^

O modelo considerou os custos diretos relacionados aos exames diagnósticos (incluindo o exame em si, taxas operacionais e insumos), bem como os custos do manejo clínico de eventos, como infarto agudo do miocárdio e hospitalização. Os valores atribuídos a cada procedimento diagnóstico foram obtidos por meio dos códigos oficiais da CBHPM e da CMED.^15,16^

Para a ACI, o custo total estimado foi de R$ 1.900,79, valor que inclui o exame, a taxa hospitalar pelo uso da sala de hemodinâmica e os custos com a monitorização do paciente durante o procedimento. Para a AngioTC o valor utilizado foi de R$ 1.311,95, conforme referência da Agência Nacional de Saúde Suplementar (ANS).

Os custos relacionados à hospitalização e ao tratamento clínico subsequente (incluindo exames complementares e medicações), em pacientes com infarto agudo do miocárdio, foram estimados com base nas tabelas oficiais disponíveis e sumarizados na Tabela S1. Os custos relacionados aos procedimentos de revascularização miocárdica cirúrgica e à angioplastia com *stent* foram obtidos conforme detalhado nas Tabelas Suplementares S2 e S3, respectivamente. Para componentes como diárias e taxas hospitalares, utilizaram-se valores médios praticados por hospitais em diferentes regiões do país, diante da ausência de uma tabela nacional padronizada. É importante destacar que esses valores costumam ser negociados diretamente entre operadoras de saúde e instituições prestadoras de serviços. Para a análise por subgrupos regionais, calcularam-se as médias de custos por região, com o objetivo de refletir as variações econômicas e estruturais observadas nas diferentes regiões do Brasil.

Para estimativa das incidências dos percentuais da probabilidade de infarto nos grupos encaminhados à AngioTC ou ACI, da probabilidade de revascularização miocárdica nos dois grupos e probabilidade de internação nos dois grupos, foram utilizadas as incidências de eventos baseadas no estudo DISCHARGE TRIAL,^17^ recente estudo multicêntrico randomizado, que comparou as estratégias AngioTC e ACI, durante seguimento médio de 3,5 anos. A Tabela S4 sumariza as probabilidades de eventos de infarto agudo do miocárdio, revascularização e hospitalizações identificadas no estudo original e aplicadas na presente análise.

Para garantir a robustez dos resultados, adicionalmente, foi conduzida uma análise de sensibilidade probabilística multivariada, por meio de simulações de coorte de Monte Carlo de segunda ordem, utilizando 1.000 interações com base nos parâmetros definidos no modelo. Os resultados dessa análise estão representados na Figura Central. Para os parâmetros com distribuição contínua e ilimitada superiormente, como os custos, foram aplicadas distribuições do tipo gama. Já para parâmetros restritos ao intervalo entre 0 e 1, como as probabilidades de eventos adversos, adotaram-se distribuições beta. As distribuições beta e gama foram parametrizadas com intervalos de variação entre 80% e 120% dos valores determinísticos, permitindo avaliar a influência de variações de até 20% sobre os resultados do modelo.

Além da análise de custo-efetividade, foi conduzido a análise de impacto orçamentário referente à incorporação da AngioTC em pacientes com suspeita de DAC estável, utilizando como população elegível estimada em 100.000 beneficiários no primeiro ano e um aumento anual proporcional à taxa de crescimento demográfico da população brasileira em um horizonte de 5 anos. Esse número de vidas buscou refletir o esperado de uma operadora de saúde de porte médio e, por consequência, a realidade do sistema de saúde suplementar atual.

A implantação da estratégia com AngioTC foi modelada de forma conservadora, em conformidade com diretrizes do Ministério da Saúde, considerando uma implementação progressiva ao longo de 5 anos. No primeiro ano, estimou-se que 5% da população elegível (5.000 indivíduos) seriam submetidos à AngioTC. A partir daí, adotou-se um incremento anual de 5 pontos percentuais, alcançando 25% da população elegível ao final do quinto ano. Essa proporção corresponde a 25.608 indivíduos, considerando a projeção de crescimento populacional derivada das estimativas demográficas do IBGE (Tabela S5).

## Resultados

A avaliação de custo-efetividade demonstrou que a estratégia da AngioTC em pacientes estáveis é dominante em relação à estratégia padrão com ACI para confirmação diagnóstica em três desfechos significativos: infarto agudo do miocárdio, hospitalizações e tratamento de revascularização miocárdica, consequente ao diagnóstico de DAC com estenose significativa. A estratégia de AngioTC demonstrou uma economia de R$ 686,86 por vida para o desfecho de infarto agudo do miocárdio. Para o desfecho hospitalização, a economia com a estratégia de AngioTC atinge um valor decremental de R$ 684,41 por vida e para o desfecho revascularização miocárdica a estratégia AngioTC atinge redução de R$ 811,09 por vida (Tabela 1).

**Tabela 1:** – Análise de custo-efetividade da angiotomografia coronária em comparação com a angiografia coronária invasiva

Desfecho	Probabilidade do evento com AngioTC (%) N=1.808*	Probabilidade do evento com ACI (%) N=1.753*	Custo total (R$)	Diferença de custo (R$)	RCEI
Infarto agudo do miocárdio	38 (2,1%)	52 (3,0%)	1.540,67	–686,86	Dominante
Revascularização miocárdica	256 (14,2%)	315 (18,0%)	2.142,47	–811,09	Dominante
Hospitalizações	10 (0,2%)	3 (0,6%)	1.359,73	–684,41	Dominante

*Dados originais extraídos do estudo DISCHARGE.^17^ ACI: angiografia coronária invasiva; AngioTC: angiotomografia coronariana; RCEI: razão de custo efetividade incremental.

Os resultados da análise de custo-efetividade são corroborados pela análise de sensibilidade, uma vez que, ao avaliar o gráfico de dispersão oriundo das simulações, percebe-se que a AngioTC segue dominante nesta análise considerando-se todos os desfechos. Na Figura Central, nota-se que a grande maioria dos pontos (91,2%) caem sobre o quadrante inferior direito, que indica dominância em relação ao comparador.

Para a análise de custo-efetividade nas 5 regiões brasileiras foram considerados valores médios aplicados em cada região do país conforme recomendação CBHPM. Os resultados obtidos das comparações, demonstram que, a AngioTC, segue sendo uma estratégia custo-efetiva, mesmo quando avaliada nas particularidades de cada região do Brasil. Os resultados da avaliação de custo-efetividade, para cada região do Brasil, de forma individual, são apresentados na Tabela 2. A análise de sensibilidade probabilística por regiões está apresentada nas Figuras Suplementares.

**Tabela 2:** – Análise de custo-efetividade da angiotomografia coronária em comparação com a angiografia coronária invasiva na redução da incidência de infarto agudo do miocárdio nas 5 regiões brasileiras

Região	Custo da AngioTC (R$)	Custo da ACI (R$)	Diferença de custo (R$)	IAM – AngioTC (probabilidade do evento, %)	IAM – ACI (probabilidade do evento, %)	Diferença de probabilidade	RCEI
Sudeste	845,00	2.249,00	–1.404,00	2.100 (2,1%)	3.000 (3,0%)	0,009	Dominante
Nordeste	813,00	2.410,00	–1.597,00				Dominante
Centro-Oeste	726,00	2.540,00	–1.814,00				Dominante
Sul	636,00	1.686,00	–1.050,00				Dominante
Norte	760,00	2.050,00	–1.290,00				Dominante

ACI: angiografia coronária invasiva; AngioTC: angiotomografia coronariana; IAM: infarto agudo do miocárdio; RCEI: razão de custo efetividade incremental.

**Figura 1:**
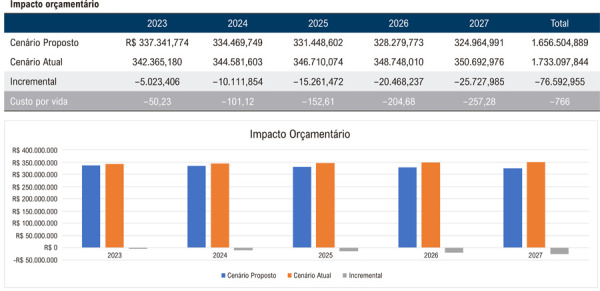
– Análise do impacto orçamentário nacional projetado para a adoção progressiva da angiotomografia coronária como estratégia diagnóstica ao longo de 5 anos. Considera-se um início com 5% de utilização no primeiro ano, com incremento anual de 5% até atingir 25% no quinto ano. São apresentados os valores estimados para o cenário atual, o cenário proposto e a diferença incremental entre ambos.

De acordo com a análise de impacto orçamentário, a implantação da estratégia da AngioTC em detrimento à ACI, com difusão da sua disponibilidade de 5% ao ano, considerando o crescimento demográfico previsto pelos dados do IBGE, resulta em uma otimização do uso de recursos projetada para população inicial de 100.000 beneficiários desde o primeiro ano de análise. A redução significativa seria de R$ 5.023.406,00 no primeiro ano, R$ 10.111.854,00 no segundo ano, R$ 15.261.472,00 no terceiro, R$ 20.468.237,00 no quarto e, finalmente, de R$ 25.727.985,00 ao final do quinto ano quando a estratégia fosse empregada para 25% da população, tendo como base o custo da AngioTC considerado 100% do valor da CBHPM (Figura 1).

**Figura 2:**
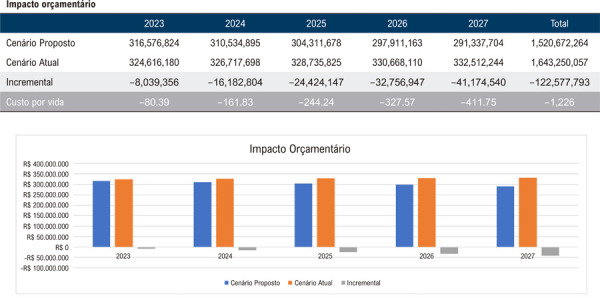
– Análise do impacto orçamentário projetado para a implementação progressiva da angiotomografia coronária como estratégia diagnóstica na Região Norte, ao longo de 5 anos. A simulação considera uma taxa inicial de adoção de 5% no primeiro ano, com aumento anual de 5%, atingindo 25% no quinto ano. São apresentados os valores estimados para o cenário atual, o cenário proposto e a diferença incremental entre ambos.

**Figura 3:**
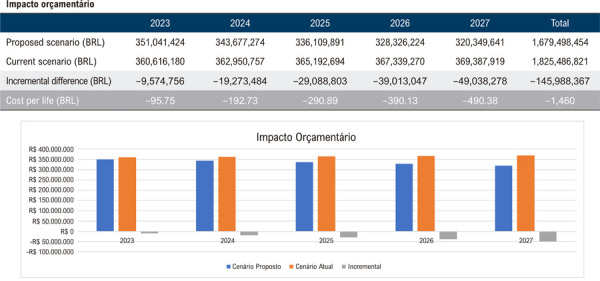
– Análise do impacto orçamentário projetado para a implementação progressiva da angiotomografia coronária como estratégia diagnóstica na Região Nordeste, ao longo de 5 anos. A simulação considera uma taxa inicial de adoção de 5% no primeiro ano, com aumento anual de 5%, atingindo 25% no quinto ano. São apresentados os valores estimados para o cenário atual, o cenário proposto e a diferença incremental entre ambos.

**Figura 4:**
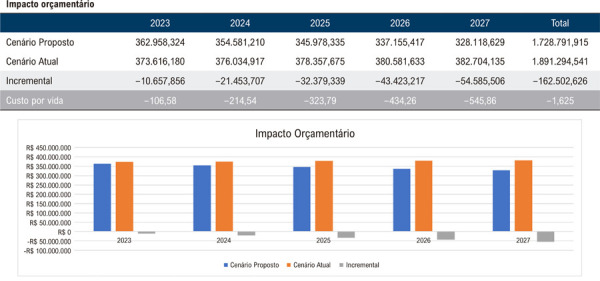
– Análise do impacto orçamentário projetado para a implementação progressiva da angiotomografia coronária como estratégia diagnóstica na Região Centro-Oeste, ao longo de 5 anos. A simulação considera uma taxa inicial de adoção de 5% no primeiro ano, com aumento anual de 5%, atingindo 25% no quinto ano. São apresentados os valores estimados para o cenário atual, o cenário proposto e a diferença incremental entre ambos.

**Figura 5:**
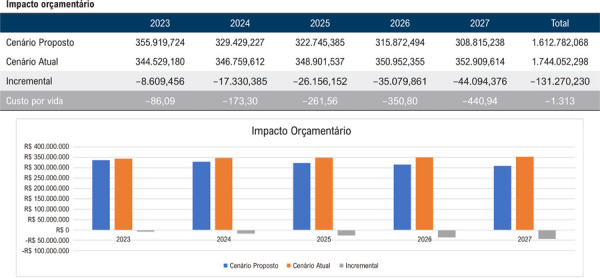
– Análise do impacto orçamentário projetado para a implementação progressiva da angiotomografia coronária como estratégia diagnóstica na Região Sudeste, ao longo de 5 anos. A simulação considera uma taxa inicial de adoção de 5% no primeiro ano, com aumento anual de 5%, atingindo 25% no quinto ano. São apresentados os valores estimados para o cenário atual, o cenário proposto e a diferença incremental entre ambos.

**Figura 6:**
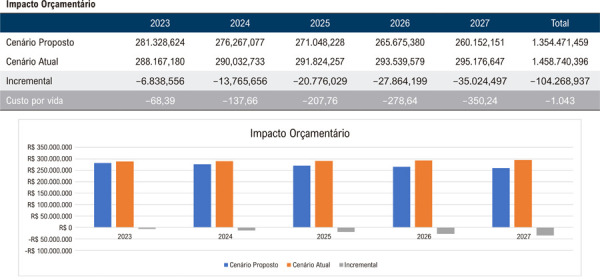
– Análise do impacto orçamentário projetado para a implementação progressiva da angiotomografia coronária como estratégia diagnóstica na Região Sul, ao longo de 5 anos. A simulação considera uma taxa inicial de adoção de 5% no primeiro ano, com aumento anual de 5%, atingindo 25% no quinto ano. São apresentados os valores estimados para o cenário atual, o cenário proposto e a diferença incremental entre ambos.

Assim, quando avaliadas as regiões de forma individual, os resultados projetam uma economia média de R$ 1.226,00 por beneficiário, totalizando R$ 122.577.793,00 ao final do quinto ano para uma população de 100.000 vidas na Região Norte, considerando a implementação da AngioTC em 25% da população elegível no último ano do horizonte temporal (Figura 2). De maneira análoga, estimou-se uma economia de R$ 1.460,00 por vida ou R$ 145.988.367,00 ao final de 5 anos na Região Nordeste (Figura 3); R$ 1.625,00 por vida ou R$ 162.502.626,00 na Região Centro-Oeste (Figura 4); R$ 1.313,00 por vida ou R$ 131.270.230,00 na Região Sudeste (Figura 5); e, por fim, R$ 1.043,00 por vida, totalizando R$ 104.268.937,00 na Região Sul (Figura 6), sempre considerando uma coorte de 100.000 indivíduos e a mesma taxa de adoção progressiva da AngioTC ao longo de 5 anos.

## Discussão

No nosso estudo, a estratégia de utilização da AngioTC como método diagnóstico inicial em pacientes estáveis com suspeita de DAC e probabilidade pré-teste intermediária demonstrou superioridade econômica em relação à ACI, tradicionalmente utilizada como padrão-ouro para diagnóstico anatômico nessa população. A análise de custo-efetividade revelou que a AngioTC é uma estratégia dominante, ou seja, mais eficaz e menos onerosa, nos desfechos clínicos avaliados: infarto agudo do miocárdio, hospitalizações e necessidade de revascularização miocárdica. Essa dominância foi consistente nas análises de sensibilidade, com 91,2% das simulações reforçando a superioridade econômica da AngioTC. Além disso, os dados por região do Brasil demonstraram robustez da estratégia mesmo frente às variações regionais de custos, mantendo a AngioTC como custo-efetiva nos cenários avaliados. A análise de impacto orçamentário projeta uma expressiva economia acumulada para uma operadora de porte médio no país. O emprego da AngioTC gera uma economia de R$ 776,00 por vida ou uma redução de R$ 25.727.985,00 ao final do quinto ano quando a estratégia fosse empregada para 25% da população.

A utilização da AngioTC tem crescido nas últimas décadas, em virtude das inúmeras evidências científicas que reforçam seu emprego na estratificação da doença coronária. Estudos utilizando AngioTC na investigação inicial diagnóstica de dor torácica em pacientes estáveis em comparação com a estratégia habitual, que utiliza fatores de risco e sintomas e/ou exames funcionais (teste ergométrico/cintilografia miocárdica), demonstraram não inferioridade da AngioTC em relação a exames funcionais, no desfecho morte cardiovascular^18^ e diminuição da incidência de infartos não fatais em até 40%, com resultados que persistiram por 10 anos de seguimento.^19^ Esses dados levaram as recentes diretrizes europeias, estadunidense e brasileira a elevarem o nível de recomendação da AngioTCc como classe I, nível de evidência A, na investigação inicial de dor torácica estável.^12,20^

Uma metanálise publicada recentemente, que incluiu estudos comparando a estratégia diagnóstica para DAC estável utilizando a AngioTC e a ACI, exibiu não inferioridade da estratégia da AngioTC no desfecho de mortalidade cardiovascular e infarto não fatal, porém, com menos revascularização miocárdica e com menor incidência de acidente vascular cerebral. Além disso, a metanálise mostrou que utilizar a estratégia de AngioTC preferencialmente permitiria evitar a necessidade de ACI em até 77% dos pacientes.^21^

Apesar das crescentes evidências das vantagens da investigação da dor torácica estável com AngioTC, com a incorporação desta estratégia nas principais diretrizes mundiais, ainda não tínhamos um estudo de custo-efetividade na saúde suplementar brasileira que incluísse AngioTC como estratégia inicial comparada com a ACI. Apesar da heterogeneidade dos valores de exames diagnósticos, hospitalização e tratamento praticados pelas diversas operadoras de saúde no Brasil, balizamos os valores utilizados para o cálculo de custo-efetividade considerando 100% dos valores recomendados pelas últimas atualizações das tabelas CBHPM e CMED. Considerando-se o cálculo de implantação desta “nova” tecnologia, seguindo recomendação conservadora e progressiva do Ministério da Saúde que recomenda iniciar o uso em somente 5% da população, aumentando progressivamente 5% ao ano em um horizonte de 5 anos, o nosso estudo mostrou que a estratégia de AngioTC é dominante e custo-efetiva, com os valores praticados na saúde suplementar brasileira.

Segundo a ANS, 24,5% da população brasileira possuía acesso à saúde suplementar em janeiro de 2025, cerca de 52 milhões de brasileiros.^22^ Segundo esta mesma fonte, cerca de 26% dos usuários de saúde suplementar estão acima de 40 anos, ou seja, estariam, devido à idade, mais propícios a ter doença aterosclerótica coronariana, com a ressalva que mulheres teriam maior prevalência de aterosclerose coronariana 10 anos após a climatério.^23^ Desta forma, se em 5 anos, em uma população de 100.000 vidas, foi demonstrada custo-efetividade utilizando AngioTC em uma fração desta população, é provável que, se a estratégia fosse disponibilizada para as milhões de pessoas com potencial risco de aterosclerose coronariana que utilizam a saúde suplementar, tivéssemos um impacto econômico ainda mais significativo.

Há 10 anos, o órgão de qualidade e decisão baseada em evidência do serviço de saúde pública do Reino Unido (NICE), recomendou a adoção da AngioTC como teste diagnóstico preferencial para investigação de dor torácica em todo o seu território.^24^ Depois de mais de meia década de experiência, os resultados dessa decisão foram apresentados e se revelaram extremamente positivos naquelas regiões do Reino Unido que conseguiram, de fato, adotar as referidas recomendações, resultando em redução significativa da mortalidade cardiovascular e uma tendência de redução na mortalidade por todas as causas.^25^ Parece razoável citar uma importante premissa em medicina, de que o diagnóstico precoce como principal estratégia permite resultados melhores, com grande economia na análise do impacto orçamentário. O paciente com diagnóstico precoce de DAC pode receber tratamento medicamentoso adequado e reduzir eventos futuros por efetivamente tratar a doença aterosclerótica.^9,26^

### Limitações

Este estudo apresenta algumas limitações importantes que devem ser consideradas na interpretação de seus resultados. Primeiramente, a modelagem de custo-efetividade foi baseada exclusivamente nos dados do estudo DISCHARGE, o que pode limitar a generalização dos achados, uma vez que os resultados de um único ensaio clínico randomizado, ainda que robusto, não contemplam todas as variabilidades clínicas e populacionais da prática cotidiana. Em segundo lugar, a análise foi restrita a pacientes com probabilidade pré-teste intermediária de DAC, o que impossibilita extrapolações para indivíduos com baixa ou alta probabilidade pré-teste, limitando sua aplicabilidade universal. Por fim, o modelo adotado compara a AngioTC diretamente com a ACI, sem considerar comparações com testes funcionais amplamente utilizados na prática clínica, como cintilografia miocárdica ou ecocardiograma com estresse, os quais continuam sendo recomendados nas diretrizes atuais para avaliação funcional da isquemia miocárdica. A ausência dessa comparação impede uma análise mais abrangente sobre o posicionamento da AngioTC frente a todas as opções diagnósticas disponíveis.

## Conclusão

A escolha da AngioTC como estratégia padrão para o diagnóstico de DAC estável parece ser custo-efetiva quando comparada à realização da ACI, no sistema de saúde suplementar brasileiro. O impacto orçamentário da implementação dessa intervenção no Brasil resultaria em redução de custos significativos para a saúde suplementar.

## Material suplementar

Suplemento
