# Supplementary material for: Análise de Custo-Efetividade da Angiotomografia Coronária como Exame Preferencial na Investigação de Dor Torácica Estável na Saúde Suplementar no Brasil
Source: Arq Bras Cardiol. 2026 Jan 9;122(12):e20250204. [Article in Portuguese] doi: 10.36660/abc.20250204 (PMC12978369; doi:10.36660/abc.20250204)

# Suplemento

## TABELAS

### Tabela S1. Valores do tratamento clínico de pacientes com infarto agudo do miocárdio.

**Custo tratamento clínico (48h)**

| | % em uso | Quantidade | Custo unitário | Custo total | FONTE |
|---|---|---|---|---|---|
| AAS (100 mg/dia) | 100% | 100 mg/dia | R$ 0,15 | R$ 0,29 | CMED/CBHPM |
| Clopidogrel (75 mg/dia) | 100% | 75 mg/dia | R$ 2,23 | R$ 4,47 | CMED/CBHPM |
| Enoxaparina (1 mg/kilo) (60mg 2xdia) | 100% | 1mg/kg - 120 mg/dia | R$ 24,93 | R$ 59,83 | CMED/CBHPM |
| Betabloqueador (atenolol ou metoprolol) (50mg/dia) | 100% | 50 mg/dia | R$ 0,32 | R$ 0,64 | CMED/CBHPM |
| Eletrocardiograma 1x/dia | 100% | 2 | R$ 69,09 | R$ 138,18 | CMED/CBHPM |
| Isossorbida sublingual (15 mg) | 100% | 15 mg/dia | R$ 1,33 | R$ 2,66 | CMED/CBHPM |
| Ultrassom ecocardiograma (1) | 100% | 1 vez ao dia | R$ 1.779,98 | R$ 1.779,98 | CMED/CBHPM |
| Rosuvastatina (20mg/dia) | 100% | 1 vez ao dia | R$ 3,32 | R$ 6,65 | CMED/CBHPM |
| Captopril (25mg 3xdia) | 100% | 75 mg/dia | R$ 0,21 | R$ 1,25 | CMED/CBHPM |
| Coagulograma (1 dosagem) | 100% | 1 | R$ 67,68 | R$ 67,68 | CMED/CBHPM |
| Colesterol Total e fracionado (1 dosagem) | 100% | 1 | R$ 65,24 | R$ 65,24 | CMED/CBHPM |
| Triglicerídeos (1 dosagem) | 100% | 1 | R$ 91,14 | R$ 91,14 | CMED/CBHPM |
| Hemograma (1 dosagem) | 100% | 1 | R$ 23,86 | R$ 23,86 | CMED/CBHPM |
| Magnésio (1 dosagem/dia UTI) | 100% | 3 | R$ 10,75 | R$ 32,25 | CMED/CBHPM |
| Troponina (1 dosagem/dia UTI) | 100% | 3 | R$ 91,14 | R$ 273,41 | CMED/CBHPM |
| Creatinina (1 dosagem/dia de UTI) | 100% | 3 | R$ 10,75 | R$ 32,25 | CMED/CBHPM |
| Uréia (1 dosagem/dia de UTI) | 100% | 3 | R$ 10,75 | R$ 32,25 | CMED/CBHPM |
| Sodio (1 dosagem/dia de UTI) | 100% | 3 | R$ 10,75 | R$ 32,25 | CMED/CBHPM |
| Potássio (1 dosagem/dia de UTI) | 100% | 3 | R$ 10,75 | R$ 32,25 | CMED/CBHPM |
| Glicemia (1 dosagem/dia UTI) | 100% | 3 | R$ 10,75 | R$ 32,25 | CMED/CBHPM |
| Cálcio (1 dosagem/dia UTI) | 100% | 3 | R$ 10,75 | R$ 32,25 | CMED/CBHPM |
| Diária de hospitalização | 100% | 2 | R$ 625,10 | R$ 1.250,20 | Opnião de especialista |
| Diária de UTI (UTI II) | 100% | 3 | R$ 2.300,00 | R$ 6.900,00 | Opnião de especialista |
| TOTAL | | | R$ 5.220,97 | R$ 10.891,23 | |

### Tabela S2. Valores médios de custo de paciente submetido a cirurgia de revascularização miocárdica.

**Custo revascularização**

| | % em uso | Quantidade | Custo unitário | Custo total | |
|---|---|---|---|---|---|
| AAS (100 mg/dia) | 100% | | R$ 0.15 | R$ 1.03 | CMED/CBHPM |
| Ultrassom ecocardiograma (1) | 100% | 1 | R$ 1 779.98 | R$ 1 779.98 | CMED/CBHPM |
| Eletrocardiograma (1 por dia de UTI) | 100% | 5 | R$ 69.09 | R$ 345.46 | CMED/CBHPM |
| Cineangiocoronariografia (1) | 100% | 1 | R$ 1 311.95 | R$ 1 311.95 | CMED/CBHPM |
| Troponina (1 por dia de UTI) | 100% | 5 | R$ 91.14 | R$ 455.68 | CMED/CBHPM |
| Hemograma (1 por dia de UTI) | 100% | 5 | R$ 23.86 | R$ 119.32 | CMED/CBHPM |
| Diaria de UTI II adulto (UTI II) (5 dias) | 100% | 5 | R$ 2 300.00 | R$ 11 500.00 | Opnião de especialista |
| Diaria de hospitalização (2 diárias) | 100% | 2 | R$ 625.10 | R$ 1 250.20 | Opnião de especialista |
| Revascularização do miocárdio c/ uso de extracorpórea* | 100% | 1 | R$ 16 048.93 | R$ 16 048.93 | CMED/CBHPM |
| Creatinina (1 dosagem/dia de UTI) | 100% | 5 | R$ 10.75 | R$ 53.75 | CMED/CBHPM |
| Uréia (1 dosagem/dia de UTI) | 100% | 5 | R$ 10.75 | R$ 53.75 | CMED/CBHPM |
| Sodio (1 dosagem/dia de UTI) | 100% | 5 | R$ 10.75 | R$ 53.75 | CMED/CBHPM |
| Potássio (1 dosagem/dia de UTI) | 100% | 5 | R$ 10.75 | R$ 53.75 | CMED/CBHPM |
| Coagulograma (1 dosagem) | 100% | 1 | R$ 67.68 | R$ 67.68 | CMED/CBHPM |
| Colesterol Total e fracionado (1 dosagem) | 100% | 1 | R$ 65.24 | R$ 65.24 | CMED/CBHPM |
| Isossorbida sublingual (15 mg) | 100% | | R$ 1.33 | R$ 9.30 | CMED/CBHPM |
| Glicemia (1 dosagem/dia UTI) | 100% | 5 | R$ 10.75 | R$ 53.75 | CMED/CBHPM |
| Cálcio (1 dosagem/dia UTI) | 100% | 5 | R$ 10.75 | R$ 53.75 | CMED/CBHPM |
| TOTAL | | | | R$ 33 277.29 | |

**Tabela S3. Valores médios de custo de paciente submetido a angioplastia com Stent.**

Custo angioplastia com stent

| | % em uso | Quantidade | Custo unitário | Custo total | |
|---|---|---|---|---|---|
| AAS (100 mg/dia) | 100% | 1 | R$ 0.15 | R$ 0.29 | CMED/CBHPM |
| Enoxaparina (1 mg/kilo) (60mg 2xdia) | 100% | 1 | R$ 24.93 | R$ 59.83 | CMED/CBHPM |
| Ultrassom ecocardiograma (1) | 100% | 1 | R$ 1 779.98 | R$ 1 779.98 | CMED/CBHPM |
| Eletrocardiograma (1 por dia de UTI) | 100% | 1 | R$ 69.09 | R$ 69.09 | CMED/CBHPM |
| Stent farmacológico | 100% | 1 | R$ 5 325.05 | R$ 5 325.05 | CMED/CBHPM |
| Diaria de UTI II adulto (UTI II) (1 dias) | 100% | 1 | R$ 2 300.00 | R$ 2 300.00 | Opinião de especialista |
| Diaria de hospitalização (1 diárias) | 100% | 1 | R$ 625.10 | R$ 625.10 | Opinião de especialista |
| Cineangiocoronariografia (1) | 100% | 1 | R$ 1 311.95 | R$ 1 311.95 | CMED/CBHPM |
| Clopidogrel (300mg ataque) (75 mg/dia) | 100% | | R$ 2.23 | R$ 13.38 | CMED/CBHPM |
| Atenolol (50mg/dia) | 100% | | R$ 0.32 | R$ 0.64 | CMED/CBHPM |
| Captopril (25mg 3xdia) | 100% | | R$ 0.21 | R$ 0.42 | CMED/CBHPM |
| Rosuvastatina (20mg/dia) | 100% | | R$ 3.32 | R$ 6.64 | CMED/CBHPM |
| Troponina (3 dosagens) | 100% | 3 | R$ 10.75 | R$ 32.25 | CMED/CBHPM |
| Hemograma (1 dosagem) | 100% | 1 | R$ 91.14 | R$ 91.14 | CMED/CBHPM |
| Creatinina (1 dosagem/dia de UTI) | 100% | 1 | R$ 91.14 | R$ 91.14 | CMED/CBHPM |
| Uréia (1 dosagem/dia de UTI) | 100% | 1 | R$ 10.75 | R$ 10.75 | CMED/CBHPM |
| Sodio (1 dosagem/dia de UTI) | 100% | 1 | R$ 10.75 | R$ 10.75 | CMED/CBHPM |
| Potássio (1 dosagem/dia de UTI) | 100% | 1 | R$ 10.75 | R$ 10.75 | CMED/CBHPM |
| Coagulograma (1 dosagem) | 100% | 1 | R$ 1 779.98 | R$ 1 779.98 | CMED/CBHPM |
| Colesterol Total e fracionado (1 dosagem) | 100% | 1 | R$ 113.13 | R$ 113.13 | CMED/CBHPM |
| Isossorbida sublingual (15 mg) | 100% | 1 | R$ 1.33 | R$ 1.33 | CMED/CBHPM |
| Glicemia (1 dosagem/dia UTI) | 100% | 1 | R$ 10.75 | R$ 10.75 | CMED/CBHPM |
| Cálcio (1 dosagem/dia UTI) | 100% | 1 | R$ 10.75 | R$ 10.75 | CMED/CBHPM |
| TOTAL | | | | R$ 13 655.09 | |

**Tabela S4. Probabilidade baseada nos dados do estudo DISCHARGE, 2022.**

| | Valor | Valor customizado | Valor utilizado | Referência |
|---|---|---|---|---|
| **Probabilidade de infarto - angio TC** | 2,10% | | 2,10% | DISCHARGE, 2022 |
| **Probabilidade de infarto - ACI** | 3,00% | | 3,00% | DISCHARGE, 2022 |
| **Probabilidade de revascularização - angio TC** | 14,20% | | 14,20% | DISCHARGE, 2022 |
| **Probabilidade de revascularização - ACI** | 18,00% | | 18,00% | DISCHARGE, 2022 |
| **Probabilidade de hospitalização - angio TC** | 0,20% | | 0,20% | DISCHARGE, 2022 |
| **Probabilidade de hospitalização - ACI** | 0,60% | | 0,60% | DISCHARGE, 2022 |

Angio-TC: Angiotomografia coronária. ACI: Angiografia coronária invasiva.

**Tabela S5. Estimativa de pacientes elegíveis considerando-se o cenário atual e o cenário proposto**

| **Cenário Atual** | **2023** | **2024** | **2025** | **2026** | **2027** |
|---|---|---|---|---|---|
| Angio-TC | - | - | - | - | - |
| ACI | 100.000 | 100.647 | 101.269 | 101.864 | 102.432 |
| **Cenário Proposto** | **2023** | **2024** | **2025** | **2026** | **2027** |
| Angio-TC | 5.000 | 10.065 | 15.190 | 20.373 | 25.608 |
| ACI | 95.000 | 90.583 | 86.079 | 81.491 | 76.824 |

Angio-TC, angiotomografia coronariana; ACI, angiografia coronariana invasiva.

Figuras Suplemento

Custo-efetividade na região Centro-Oeste

| Desfechos | Angiotomografia computadorizada (angio-TC) | ACI | Incremental |
|---|---|---|---|
| Custo total | R$ 813 | R$ 2.410 | -R$ 1.597 |
| Infarto agudo do miocárdio (IAM) | 0,021 | 0,030 | 0,009 |
| RCEI (R$) | | | Dominante |

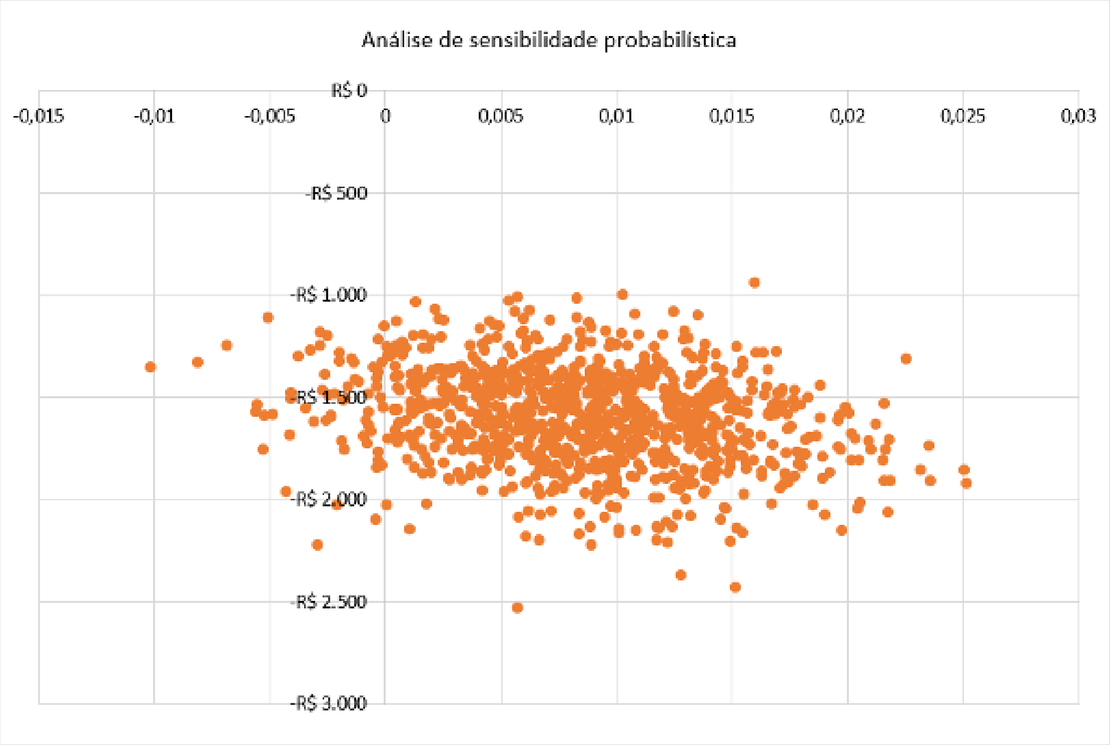

Custo-efetividade na região Norte

| Desfechos | Angiotomografia computadorizada (angio-TC) | ACI | Incremental |
|---|---|---|---|
| Custo total | R$ 760 | R$ 2.050 | -R$ 1.290 |
| Infarto agudo do miocárdio (IAM) | 0,021 | 0,030 | 0,009 |
| RCEI (R$) | | | Dominante |

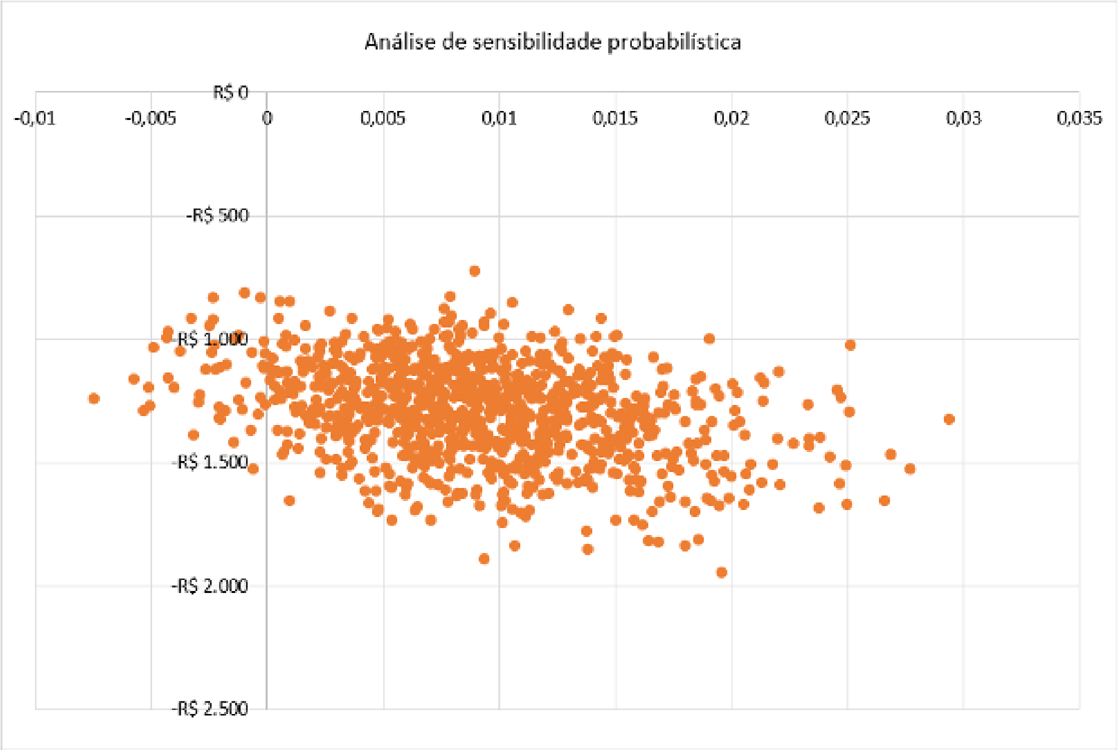

Custo-efetividade na região Nordeste

| Desfechos | Angiotomografia computadorizada (angio-TC) | ACI | Incremental |
|---|---|---|---|
| Custo total | R$ 813 | R$ 2.410 | -R$ 1.597 |
| Infarto agudo do miocárdio (IAM) | 0,021 | 0,030 | 0,009 |
| RCEI (R$) | | | Dominante |

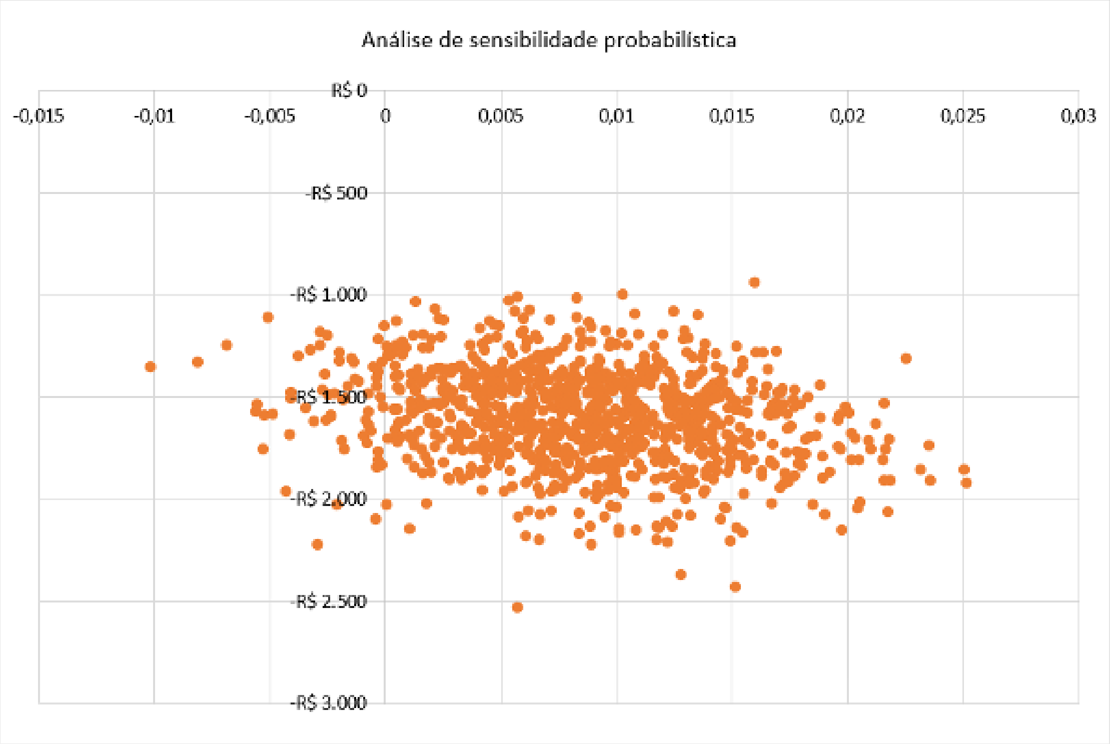

Custo-efetividade na região Sul

| Desfechos | Angiotomografia computadorizada (angio-TC) | ACI | Incremental |
|---|---|---|---|
| Custo total | R$ 636 | R$ 1.686 | -R$ 1.050 |
| Infarto agudo do miocárdio (IAM) | 0,021 | 0,030 | 0,009 |
| RCEI (R$) | | | Dominante |

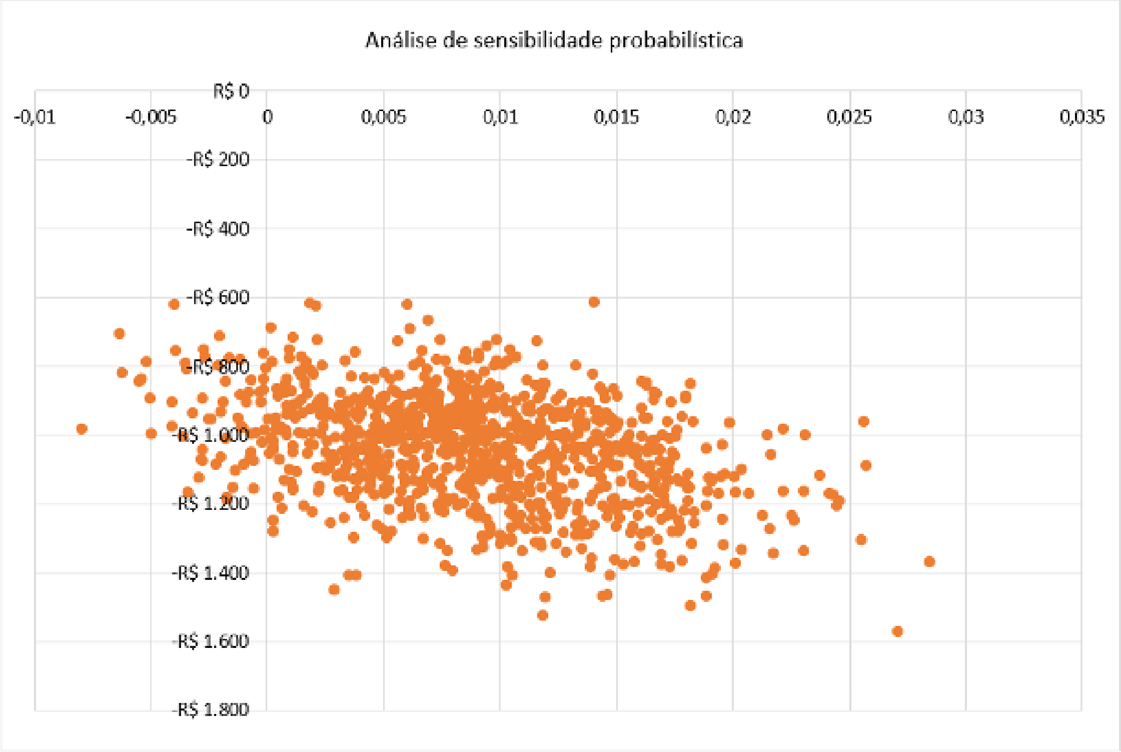

Custo-efetividade na Região Sudeste

| Desfechos | Angiotomografia computadorizada (angio-TC) | ACI | Incremental |
|---|---|---|---|
| Custo total | R$ 845 | R$ 2.249 | -R$ 1.404 |
| Infarto agudo do miocárdio (IAM) | 0,021 | 0,030 | 0,009 |
| RCEI (R$) | | | Dominante |

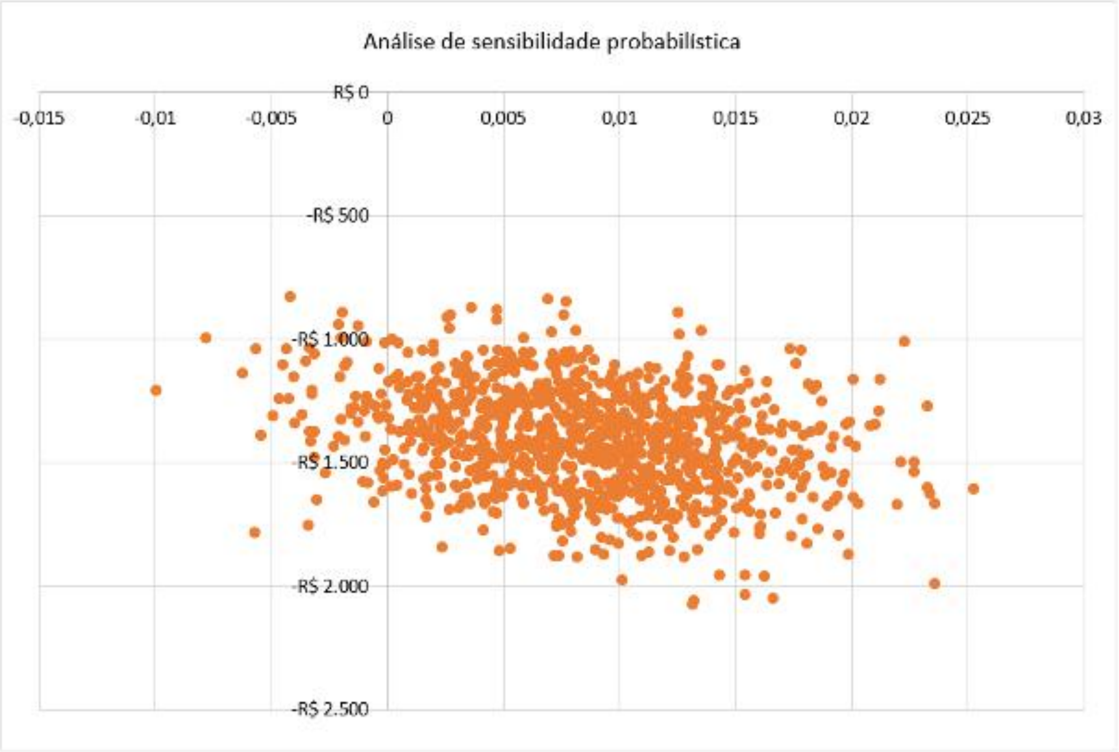

Supplement: Suplemento [file 0066-782x-abc-122-12-e20250204-suppl01.pdf]
