# Supplementary material for: Análise de Custo-Efetividade da Angiotomografia Coronária como Exame Preferencial na Investigação de Dor Torácica Estável na Saúde Suplementar no Brasil
Source: Arq Bras Cardiol. 2026 Jan 9;122(12):e20250204. [Article in Portuguese] doi: 10.36660/abc.20250204 (PMC12978369; doi:10.36660/abc.20250204)
Supplement: Translation

# Translation: Cost-Effectiveness Analysis of Coronary Computed Tomography Angiography as the Preferred Exam in the Investigation of Stable Chest Pain in the Brazilian Private Healthcare System

**DOI:** 10.36660/abc.20250204i

**Authors:** Afonso Shiozaki, Jorge Torreão, Isabela Bispo Santos da Silva Costa, Anuncia Bouzas Suarez, Marcelo Tozatti da Silva, Thiago Godoy de Oliveira, Luana Emanuelly Sinhori Lopes, Marcelo Eidi Nita, Henrique Trad, Carlos E. Rochitte

**Affiliations:** 1 Brazil ND Núcleo Diagnóstico, Maringá, PR – Brazil; 2 Brazil Santa Casa de Misericórdia da Bahia, Salvador, BA – Brazil; 3 Brazil Instituto do Câncer do Estado de São Paulo, São Paulo, SP – Brazil; 4 Brazil Fundação Getúlio Vargas, Rio de Janeiro, RJ – Brazil; 5 Brazil Universidade de Mogi das Cruzes, Mogi das Cruzes, SP – Brazil; 6 Brazil Universidade Federal do ABC, Santo André, SP – Brazil; 7 Brazil Universidade Estadual do Oeste do Paraná, Cascavel, PR – Brazil; 8 Brazil Universidade de São Paulo, São Paulo, SP – Brazil; 9 Brazil Lotus Radiologia, Ribeirão Preto, SP – Brazil; 10 Brazil Instituto do Coração do Hospital das Clínicas da Faculdade de Medicina da Universidade de São Paulo, São Paulo, SP – Brazil

**Keywords:** X-Ray Computed Tomography, Coronary Artery Disease, Cost-Effectiveness Analysis, Myocardial Infarction

## Abstract

**Background:**

Cardiovascular disease is the leading cause of mortality worldwide. Strategies that prioritize early diagnosis can reduce the incidence of related complications and cost.

**Objective:**

To assess the cost-effectiveness of coronary computed tomography angiography (CCTA) as the initial diagnostic strategy for stable chest pain in patients with intermediate pre-test probability of stable coronary artery disease (CAD), in comparison with invasive coronary angiography (ICA).

**Methods:**

A cost-effectiveness analysis was conducted comparing CCTA and ICA, considering data from the Brazilian private healthcare system. The model considered the direct costs of diagnostic exams, medical supplies, hospitalization for myocardial infarction, and myocardial revascularization in the 5 regions of Brazil. A budget impact analysis was performed regarding the gradual incorporation of CCTA over 5 years, considering 100,000 lives as the eligible population.

**Results:**

The cost-effectiveness analysis comparing CCTA to ICA, estimated for a population of 100,000 lives, demonstrated cost savings of BRL 1,021.00 per life or a total of BRL 102,069,703.00 by the end of the fifth year. When considering the regional average cost of CCTA, for a population of 100,000 over 5 years, we observed the following cost savings per life and in 5 years, respectively: BRL 1,226.00 and BRL 122,577,793.00 in the North Region; BRL 1,460.00 and BRL 145,988,367.00 in the Northeast Region; BRL 1,625.00 and BRL 162,502,626.00 in the Central-West Region; BRL 1,313.00 and BRL 131,270,230.00 in the Southeast Region; and BRL 1,043.00 and BRL 104,268,937.00 in the South Region.

**Conclusion:**

As an initial strategy for investigating stable chest pain, CCTA is cost-effective compared to ICA and is associated with significant cost reductions in the Brazilian private healthcare system.

## Introduction

Cardiovascular disease is the leading cause of death worldwide, surpassing all causes of cancer combined, killing 3 times more women than breast cancer and 7 times more than colon cancer.^1-3^ In Brazil, they are responsible for more than 350,000 deaths per year, more than 1,100 deaths per day, approximately 46 deaths per hour, or 1 death every 90 seconds.^4^ Given this high burden of mortality, it is essential to provide early diagnosis of coronary artery disease (CAD) in order to reduce complications related to major cardiovascular events.

CAD represents a significant impact on both public health systems and national economies. It is estimated that, in Brazil, cardiovascular diseases generate direct and indirect costs exceeding R$50 billion per year.^5^ These costs range from consultations, diagnostic tests, and pharmacological therapies to highly complex procedures such as angioplasty and myocardial revascularization, which have increased in recent years in Brazil.^3^ Furthermore, indirect costs related to loss of productivity, early retirement, and recurrent hospitalizations further increase the financial burden. Accordingly, investments in diagnostic strategies that can provide early and accurate diagnosis of CAD have the potential not only to improve patients’ clinical prognosis but also to mitigate potential long-term costs by enabling timely interventions that prevent adverse outcomes and avoidable hospitalizations.

The diagnostic strategy for CAD is often complex and is discussed in various clinical situations according to the presence of symptoms and cardiovascular risk factors. Invasive coronary angiography (ICA) is the gold standard method for anatomical diagnosis of CAD.^6^ However, it is an invasive method, which is not free from related complications.^7^ Coronary computed tomography angiography (CCTA) is the only non-invasive test capable of visualizing coronary atherosclerotic plaque in various phases of development, including non-obstructive ones, allowing for earlier detection and treatment of CAD and reducing the absolute number of infarctions.^8,9^ A series of results from the Ischemia Trial Group seems to have consolidated the concept that the detection and characterization of atherosclerotic burden has a prognostic value superior to the detection of myocardial ischemia.^10,11^ Current Brazilian guidelines recommend this exam as an initial diagnostic strategy in patients with chest pain and unknown anatomy.^12^ Nevertheless, there are no Brazilian data demonstrating the cost-effectiveness of this approach in the private healthcare system.

Given the above, this study conducted a cost-effectiveness analysis comparing available strategies for anatomical diagnosis of CAD (CCTA versus ICA) as the initial procedure for evaluating patients with stable angina in the Brazilian private healthcare system. The objective was to estimate the potential cost reduction and clinical impact of systematically adopting CCTA, with specific modeling for different geographic regions of Brazil in order to reflect variations in costs and available infrastructure.

## Methods

A cost-effectiveness and budget impact assessment was conducted, considering CCTA as the initial strategy for investigating CAD compared to ICA. ICA was chosen as the comparator due to the fact that it is considered the gold standard diagnostic method for CAD stratification and is widely available in the Brazilian private healthcare system. This comparison is justified by the fact that CCTA is the only non-invasive method available for anatomical diagnosis of CAD as well as its high diagnostic accuracy.^13^

With the aim of increasing the transparency of the proposed study, the main aspects of the analyses were summarized according to the Consolidated Health Economic Evaluation Reporting Standards (CHEERS) checklist, over a 5-year horizon, as recommended by the Brazilian Ministry of Health.^14^

The analysis focused on the use of CCTA as the initial diagnostic tool for patients with suspected stable CAD, in comparison with ICA. The population assessed in the model corresponds to the simulated demand of a mid-sized healthcare provider, estimated at an annual cohort of 100,000 patients. Additionally, the annual demographic growth rate defined by the Brazilian Institute of Geography and Statistics (IBGE) was applied to the population projection.

The economic assessment was conducted based on prices charged within the Brazilian private healthcare system, applying the Brazilian Hierarchical Classification of Medical Procedures (CBHPM), put forth by the Brazilian Medical Association (AMB) in conjunction with medical specialty societies, with technical support from the Economic Research Institute Foundation (FIPE), as a reference. Additionally, we used the official drug price list published by the Drug Market Regulation Chamber (CMED), an interministerial body composed of representatives from the Brazilian Ministries of Health, Economy, Justice, and the Chief of Staff, with the National Health Surveillance Agency (ANVISA) responsible for its Executive Secretariat.^15,16^

The model considered direct costs related to diagnostic tests (including the test itself, operating fees, and medical supplies), as well as the costs of clinical management of events, such as acute myocardial infarction and hospitalization. The values assigned to each diagnostic procedure were obtained using official codes from the CBHPM and CMED.^15,16^

For ICA, the total estimated cost was BRL 1,900.79, which includes the test, the hospital fee for use of the catheterization room, and the costs of patient monitoring during the procedure. For CCTA, the cost of BRL 1,311.95 was considered, according to the reference provided by the National Supplementary Health Agency (ANS), which is the regulatory body responsible for private healthcare in Brazil.

The costs related to hospitalization and subsequent clinical treatment (including additional tests and medications) for patients with acute myocardial infarction were estimated based on official tables made available and summarized in Supplementary Table S1. The costs related to surgical myocardial revascularization procedures and stent angioplasty were obtained as detailed in Supplemental Tables S2 and S3, respectively. For components such as hospital fees and daily rates, average values charged by hospitals in different regions of the country were used, given the lack of a standardized national price list. It is important to note that these values are often negotiated directly between health insurance companies and service providers. For analysis by regional subgroups, average costs were calculated by region to reflect the economic and structural variations observed in different Brazilian regions.

To estimate the incidence of the percentage probability of infarction in the groups referred for CCTA or ICA, the probability of myocardial revascularization in both groups, and the probability of hospitalization in both groups, we used the event incidences based on the DISCHARGE TRIAL study,^17^ a recent multicenter randomized trial that compared CCTA and ICA strategies over a mean follow-up of 3.5 years. Supplementary Table S4 summarizes the probabilities of acute myocardial infarction, revascularization, and hospitalization events identified in the original study and applied in the present analysis.

To guarantee the robustness of the results, a multivariate probabilistic sensitivity analysis was also conducted, by means of second-order Monte Carlo simulations, using 1,000 interactions based on the parameters defined in the model. The results of this analysis are shown in Central Illustration. For parameters with continuous and upper incomplete distribution, such as costs, gamma distributions were applied. For parameters restricted to the range between 0 and 1, such as the probabilities of adverse events, beta distributions were adopted. The beta and gamma distributions were parameterized with variation intervals between 80% and 120% of the deterministic values, allowing assessment of the influence of variations of up to 20% on the model results.

In addition to the cost-effectiveness analysis, a budget impact analysis was conducted regarding the incorporation of CCTA in patients with suspected stable CAD, using an estimated eligible population of 100,000 patients in the first year and an annual increase proportional to the demographic growth rate of the Brazilian population over a 5-year horizon. This number of lives sought to reflect the expectations of a mid-sized health insurance provider and, consequently, the reality of the current private healthcare system in Brazil.

The implementation of the CCTA strategy was modeled conservatively, in accordance with Ministry of Health guidelines, considering progressive implementation over 5 years. In the first year, it was estimated that 5% of the eligible population (5,000 individuals) would undergo CCTA. Thereafter, an annual increase of 5% was adopted, reaching 25% of the eligible population by the end of the fifth year. This proportion corresponds to 25,608 individuals, considering the population growth projection derived from IBGE demographic estimates (Table S5).

## Results

Cost-effectiveness assessment demonstrated that the CCTA strategy in stable patients was superior to the standard strategy with ICA for diagnostic confirmation in three significant outcomes: acute myocardial infarction, hospitalizations, and myocardial revascularization, as a consequence of diagnosis of CAD with significant stenosis. The CCTA strategy demonstrated savings of BRL 686.86 per life for the outcome of acute myocardial infarction. For the outcome of hospitalization, the savings with the CCTA strategy reached a decreasing value of BRL 684.41 per life, and for the outcome of myocardial revascularization, the CCTA strategy achieved a reduction of BRL 811.09 per life (Table 1).

**Table 1:** – Cost-effectiveness analysis of coronary computed tomography angiography compared with invasive coronary angiography

Outcome	Event probability with CCTA (%) N=1808*	Event probability with ICA (%) N=1753*	Total cost (BRL)	Cost difference (BRL)	ICER
Acute myocardial infarction	38 (2.1%)	52 (3.0%)	1,540.67	−686,86	Dominant
Myocardial revascularization	256 (14.2%)	315 (18.0%)	2,142.47	−811,09	Dominant
Hospitalizations	10 (0.2%)	3 (0.6%)	1,359.73	−684,41	Dominant

*Original data extracted from the DISCHARGE study.^17^ CCTA: coronary computed tomography angiography; ICA: invasive coronary angiography; ICER: incremental cost-effectiveness ratio.

The cost-effectiveness analysis results are corroborated by the sensitivity analysis, as the scatterplot from the simulations reveals that CCTA remained dominant in this analysis, considering all outcomes. Central Illustration shows that the vast majority of points (91.2%) fell in the lower right quadrant, indicating dominance over the comparator.

For cost-effectiveness analysis in the 5 Brazilian regions, average values applied in each region of the country were considered, as recommended by the CBHPM. The results obtained from the comparisons demonstrated that CCTA remained a cost-effective strategy, even when assessed within the specificities of each region of Brazil. The cost-effectiveness assessment results for each Brazilian region are individually displayed in Table 2. The probabilistic sensitivity analysis by region is shown in the Supplementary Figures.

**Table 2:** – Cost-effectiveness analysis of coronary computed tomography angiography compared with invasive coronary angiography in reducing the incidence of acute myocardial infarction by Brazilian region

Region	Cost of CCTA (BRL)	Cost of ICA (BRL)	Cost difference (BRL)	AMI with CCTA (event probability, %)	AMI with ICA (event probability, %)	Probability difference	ICER
Southeast	845.00	2,249.00	–1,404.00	2,100 (2.1%)	3,000 (3.0%)	0.009	Dominant
Northeast	813.00	2,410.00	–1,597.00				Dominant
Central-West	726.00	2,540.00	–1,814.00				Dominant
South	636.00	1,686.00	–1,050.00				Dominant
North	760.00	2,050.00	–1,290.00				Dominant

AMI: acute myocardial infarction; CCTA: coronary computed tomography angiography; ICA: invasive coronary angiography; ICER: incremental cost-effectiveness ratio.

According to budget impact analysis, implementing the CCTA strategy instead of ICA, with a 5% annual increase in availability, considering the population growth predicted by IBGE data, would result in an optimization of the use of resources projected for the initial population of 100,000 patients from the first year of analysis. The significant reduction would be BRL 5,023,406.00 in the first year, BRL 10,111,854.00 in the second year, BRL 15,261,472.00 in the third, BRL 20,468,237.00 in the fourth, and BRL 25,727,985.00 at the end of the fifth year, when employing the strategy for 25% of the population, based on the cost of CCTA considering 100% of the CBHPM value (Figure 1).

**Figure 1:** – Projected budget impact analysis for the progressive implementation of coronary computed tomography angiography as a diagnostic strategy in Brazil over 5 years. The simulation assumes an initial adoption rate of 5% in the first year, with an annual increase of 5%, reaching 25% in the fifth year. Estimated values are displayed for the current scenario, the proposed scenario, and the incremental difference between the two.

When assessing regions on an individual basis, the results for the North Region projected an average savings of BRL 1,226.00 per patient, totaling BRL 122,577,793.00 at the end of the fifth year for a population of 100,000 lives, considering the implementation of CCTA in 25% of the eligible population in the final year of the time horizon (Figure 2). Similarly, the model estimated savings of BRL 1,460.00 per life or BRL 145,988,367.00 at the end of 5 years for the Northeast Region (Figure 3); BRL 1,625.00 per life or BRL 162,502,626.00 for the Central-West Region (Figure 4); BRL 1,313.00 per life or BRL 131,270,230.00 for the Southeast Region (Figure 5); and BRL 1,043.00 per life or BRL 104,268,937.00 in total for the South Region (Figure 6). All regional analyses considered a cohort of 100,000 individuals and the same rate of progressive adoption of CCTA over 5 years.

**Figure 2:** – Projected budget impact analysis for the progressive implementation of coronary computed tomography angiography as a diagnostic strategy in the North Region of Brazil over 5 years. The simulation assumes an initial adoption rate of 5% in the first year, with an annual increase of 5%, reaching 25% in the fifth year. Estimated values are displayed for the current scenario, the proposed scenario, and the incremental difference between the two.

**Figure 3:** – Projected budget impact analysis for the progressive implementation of coronary computed tomography angiography as a diagnostic strategy in the Northeast Region of Brazil over 5 years. The simulation assumes an initial adoption rate of 5% in the first year, with an annual increase of 5%, reaching 25% in the fifth year. Estimated values are displayed for the current scenario, the proposed scenario, and the incremental difference between the two.

**Figure 4:** – Projected budget impact analysis for the progressive implementation of coronary computed tomography angiography as a diagnostic strategy in the Central-West Region of Brazil over 5 years. The simulation assumes an initial adoption rate of 5% in the first year, with an annual increase of 5%, reaching 25% in the fifth year. Estimated values are displayed for the current scenario, the proposed scenario, and the incremental difference between the two.

**Figure 5:** – Projected budget impact analysis for the progressive implementation of coronary computed tomography angiography as a diagnostic strategy in the Southeast Region of Brazil over 5 years. The simulation assumes an initial adoption rate of 5% in the first year, with an annual increase of 5%, reaching 25% in the fifth year. Estimated values are displayed for the current scenario, the proposed scenario, and the incremental difference between the two.

**Figure 6:** – Projected budget impact analysis for the progressive implementation of coronary computed tomography angiography as a diagnostic strategy in the South Region of Brazil over 5 years. The simulation assumes an initial adoption rate of 5% in the first year, with an annual increase of 5%, reaching 25% in the fifth year. Estimated values are displayed for the current scenario, the proposed scenario, and the incremental difference between the two.

## Discussion

In our study, the strategy of using CCTA as the initial diagnostic method in stable patients with suspected CAD and intermediate pre-test probability demonstrated economic superiority over ICA, traditionally used as the gold standard for anatomical diagnosis in this population. Cost-effectiveness analysis revealed CCTA as a dominant strategy, i.e., more effective and less costly, for the following clinical outcomes assessed: acute myocardial infarction, hospitalizations, and the need for myocardial revascularization. This dominance was consistent in the sensitivity analyses, with 91.2% of simulations reinforcing the economic superiority of CCTA. Furthermore, data by Brazilian region demonstrated the robustness of the CCTA strategy, even considering regional cost variations, maintaining its cost-effectiveness in the scenarios assessed. The budget impact analysis projects significant cumulative savings for a mid-sized operator in Brazil. The use of CCTA would generate savings of 776.00 BRL per life or a reduction of 25,727,985.00 BRL at the end of the fifth year when implementing the strategy for 25% of the population.

The use of CCTA has grown in recent decades, due to the large volume of scientific evidence that has reinforced its use in the stratification of CAD. Studies assessing CCTA in the initial diagnostic investigation of chest pain in stable patients compared with the standard strategy, which uses risk factors and symptoms and/or functional tests (exercise stress testing/myocardial scintigraphy), demonstrated non-inferiority of CCTA in relation to functional tests for the outcome of cardiovascular death^18^ and a reduction in the incidence of non-fatal infarctions by up to 40%, with results that persisted for 10 years of follow-up.^19^ These data led recent European, United States, and Brazilian guidelines to raise the recommendation level of CCTA to class I, level of evidence A, in the initial investigation of stable chest pain.^12,20^

A recently published meta-analysis, which compared the diagnostic strategy for stable CAD using CCTA and ICA, demonstrated non-inferiority of the CCTA strategy for the outcomes of cardiovascular mortality and non-fatal myocardial infarction, but with fewer myocardial revascularization procedures and a lower incidence of stroke. Furthermore, the meta-analysis demonstrated that preferentially using CCTA could avoid the need for ICA in 77% of patients.^21^

Despite the growing evidence of the advantages of investigating stable chest pain by means of CCTA, with the incorporation of this strategy into major global guidelines, there were no cost-effectiveness studies in the context of Brazilian private healthcare that included CCTA as an initial strategy compared to ICA. Despite the heterogeneity in costs of diagnostic tests, hospitalization, and treatment charged by different health insurance providers in Brazil, we based the values used to calculate cost-effectiveness on 100% of those recommended by the most recent updates of the CBHPM and CMED tables. Considering the calculation of implementing this “new” technology, following the conservative progressive recommendation of the Ministry of Health, which recommends starting use in only 5% of the population, progressively increasing 5% per year over a 5-year horizon, our study showed that the CCTA strategy is dominant and cost-effective, considering the values practiced in the Brazilian private healthcare system.

According to the ANS, 24.5% of the Brazilian population had access to private healthcare in January 2025, corresponding to approximately 52 million Brazilians.^22^ According to the same source, approximately 26% of private healthcare users are over 40 years old, meaning they would, due to their age, be more likely to have coronary atherosclerotic disease, also taking into account the fact that women have a higher prevalence of coronary atherosclerosis 10 years after menopause.^23^ Thus, if in 5 years, in a population of 100,000 lives, cost-effectiveness was demonstrated using CCTA in a fraction of this population, it is likely that, were the strategy made available to the millions of people at potential risk of coronary atherosclerosis who use private healthcare, the economic impact would be even more significant.

Ten years ago, in the United Kingdom, the National Institute for Health and Care Excellence (NICE) recommended the adoption of CCTA as the preferred diagnostic test for investigating chest pain throughout the territory.^24^ After more than half a decade of experience, the results of this decision were presented, and they proved extremely positive in the regions of the United Kingdom that were able to effectively adopt the recommendations, resulting in a significant reduction in cardiovascular mortality and a downward trend in all-cause mortality.^25^ It seems reasonable to cite an important premise in medicine, namely, that early diagnosis as the primary strategy allows for better outcomes, with significant savings in budget impact analysis. Patients with early diagnosis of CAD can receive appropriate drug treatment and reduce future events by effectively treating atherosclerotic disease.^9,26^

### Limitations

There are some important study limitations that should be considered when interpreting these findings. First, the cost-effectiveness model was based exclusively on data from the DISCHARGE study, which may limit the generalizability of the findings, seeing that the results of a single randomized clinical trial, while robust, do not encompass all clinical and population variability in daily practice. Second, the analysis was restricted to patients with intermediate pre-test probability of CAD, making it impossible to extrapolate to patients with low or high pre-test probability, thus limiting universal applicability. Finally, the adopted model compares CCTA directly with ICA, without considering comparisons with functional tests that are widely used in clinical practice, for example, myocardial scintigraphy or stress echocardiography, which are still recommended in current guidelines for functional assessment of myocardial ischemia. The absence of this comparison prevents a more comprehensive analysis of the positioning of CCTA compared to all available diagnostic options.

## Conclusion

The choice of CCTA as the standard strategy for diagnosing stable CAD appears to be cost-effective when compared to ICA in the context of the Brazilian private healthcare system. The budget impact of implementing this intervention in Brazil would result in significant cost savings for private healthcare.

## *Supplemental Materials

Supplementary material
